# Bioequivalence of Sandoz methylphenidate osmotic-controlled release tablet with Concerta® (Janssen-Cilag)

**DOI:** 10.1002/prp2.72

**Published:** 2015-01-05

**Authors:** Elisabeth Schapperer, Heike Daumann, Stéphane Lamouche, Ursula Thyroff-Friesinger, François Viel, Werner Weitschies

**Affiliations:** 1 Sandoz Development Center HolzkirchenHolzkirchen, Germany; 2 inVentiv Health ClinicalMontréal, Quebec, Canada; 3 Center of Drug Absorption and Transport, Institute of Pharmacy, University of GreifswaldGreifswald, Germany

**Keywords:** ADHD, bioequivalence, Concerta®, extended release, methylphenidate, osmotic-controlled release, Sandoz

## Abstract

The aim was to assess the bioequivalence of Sandoz methylphenidate osmotic-controlled release (OCR) tablets (Sandoz [Methylphenidate[ MPH OCR) with Concerta®, a methylphenidate formulation indicated for the treatment of attention deficit/hyperactivity disorder (ADHD). Four open-label, randomized, single-dose, two-way crossover bioequivalence studies were conducted in healthy subjects: three fasting studies with 54-, 36- and 18-mg doses of methylphenidate, and one fed study with the 54-mg dose. The d- and l-threo-methylphenidate plasma levels were quantified using liquid chromatographic methods with tandem mass spectrometry (LC MS/MS). Bioequivalence of the formulations was accepted if the 90% geometric confidence intervals of the ratio of least-squares means of Sandoz MPH OCR to Concerta® of ln-transformed area under the curve (AUC_0–*t*_) and *C*_max_ were within the acceptance range of 80–125%. All studies met the bioequivalence criteria, and 90% geometric confidence intervals for AUC_0–*t*_ and *C*_max_ were within the predefined range. All plasma concentration time curves for Sandoz MPH OCR under fasting conditions showed a biphasic profile comparable with Concerta®, confirmed by bioequivalence of the partial metrics AUC_0–2h_, AUC_2-24 h_, *C*_max(0–2 h)_ and *C*_max(2–24 h)_. Both products were well tolerated and no relevant differences in the safety profiles were observed. It was concluded that Sandoz MPH OCR is bioequivalent to Concerta® in terms of rate and extent of absorption when administered as a single dose of one extended-release tablet of 54, 36, or 18 mg under fasting conditions and at a dose of 54 mg under fed conditions.

## Introduction

Attention deficit hyperactivity disorder (ADHD) is a common neurobehavioural disorder which causes impairment in multiple domains of everyday functioning (Weisler and Childress 2011). Estimates of prevalence range from 1.7 to 17.8% depending on the population assessed and the diagnostic criteria applied (Markowitz et al. 2003). ADHD first presents in childhood and for many it persists into adulthood (Markowitz et al. 2003). Methylphenidate (MPH), a psychostimulant affecting both the dopaminergic and the noradrenergic systems, has an established safety and efficacy profile and is one of the most frequently prescribed treatments for ADHD in children and adults (Markowitz et al. 2003; Engert and Pruessner 2008; Janssen-Cilag 2013). It is available in both immediate-release formulations, which release MPH quickly upon ingestion, and extended-release formulations, which release some or all of the active substance more slowly over several hours. (Markowitz et al. 2003).

Concerta® (Janssen-Cilag), indicated for the treatment of ADHD in children and adults aged 6–65 years, is an extended-release formulation that has an immediate-release portion of 22% of the total drug load in a water soluble outer coating and an extended-release core that delivers 78% of the MPH dose. The extended-release core is an osmotic controlled-release oral delivery system (OROS) which is surrounded by the IR overcoat (López and Leroux 2013). Sandoz has developed a generic osmotic-controlled release (OCR) methylphenidate formulation (Sandoz MPH OCR) to be bioequivalent to Concerta®.

The Concerta® formulation delivers a biphasic plasma concentration curve for MPH with two phases of drug release. The first phase is determined by the immediate-release dose fraction, which provides a therapeutic drug level shortly after administration and is thought to prevent the development of acute tolerance to MPH (Markowitz et al. 2003; Swanson et al. 2003). The second phase provides the dose of MPH required to maintain an effective therapeutic level for a prolonged period (Markowitz et al. 2003; Swanson et al. 2003). The European Medicines Agency (EMA) guidelines (The European Medicines Agency 1999) in general require bioequivalence studies under fasting, fed, and multiple doses for a generic modified-release product. In the case of Sandoz MPH OCR, a multiple-dose study was not required as no drug accumulation occurs (Sandoz 2011). Furthermore, for biphasic products, bioequivalence must be established in both the immediate- and extended-release phases (The European Medicines Agency 2013). In vivo bioequivalence is almost always established in healthy adult volunteers and this is regarded as adequate for extrapolation to all populations in which the drug is approved, including both adults and children (EMA CPMP/QWP/EWP/1401/98 Rev. 1, 2010).

It was the goal of the studies to assess bioequivalence, compare the rate and extent of absorption, and characterize the pharmacokinetic profile of Sandoz MPH OCR relative to Concerta® in healthy adult human male subjects at 54-, 36- and 18-mg doses under fasting conditions, and with a 54-mg dose under fed conditions. Furthermore, the safety of the formulations was also assessed during the study program on the basis of clinical and laboratory examinations and adverse events were documented.

## Materials and Methods

### Trial design

**Figure 1:**
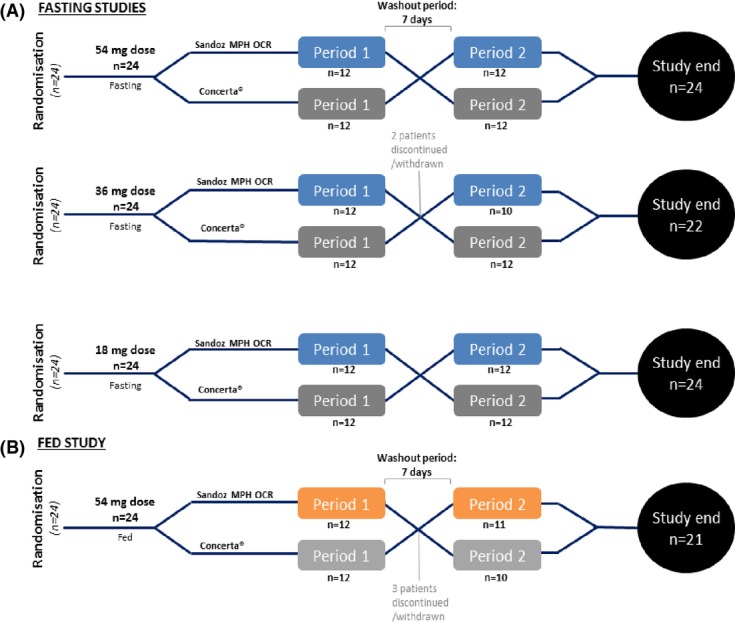
Design of the four bioequivalence studies.

Four open-label, randomized, single-dose, two-way crossover bioequivalence studies were conducted between November 2009 and April 2010 at a single centre in Québec, Canada. The duration of the clinical part of each trial was approximately 9 days (approximately 10 h prior to the dose administration in Period I until 24 h after the administration of the product in Period II). This included a washout period of 7 days (at least 10 times the mean half-life of methylphenidate hydrochloride [Janssen-Cilag 2013[), which was chosen to allow the complete elimination of the drug before subsequent dosing and to avoid carry-over effects (Fig.1). A total of 22 blood samples were collected prior to drug administration and up to 24 h post-dose, in each period, in each study.

Three studies were conducted under fasting conditions with MPH doses of 54, 36 and 18 mg, respectively; the fourth study was conducted under fed conditions with a dose of 54 mg. The pharmacokinetic profile of Sandoz MPH OCR was characterized relative to Concerta®, and bioequivalence was assessed. For the fasting studies, subjects were fasted for at least 10 h before dosing and were served a controlled meal not less than 4 h post-dose and at appropriate times thereafter, in each period. For the 54-mg dose fed study, after a supervised overnight fast of at least 10 h, and 30 min before drug administration, subjects were served a high-fat, high-caloric breakfast of ∼800–1000 calories, which was in line with EMA and FDA guidelines (The US Food and Drug Administration 2002). The breakfast consisted of two eggs fried in butter, two slices of toast with butter, two strips of bacon, approximately 128 g of hash brown potatoes, and 200 mL of whole milk (3.25% milk fat). Subsequent to dosing, subjects were served a controlled meal not less than 4 h post-dose, and at appropriate times thereafter, in each period.

### Study subjects

Healthy Caucasian male volunteers were enrolled; all were aged between 18 and 55 years, were non-smokers and had a Body Mass Index (BMI) of 19–27 kg/m^2^, with no significant illness or clinically significant abnormal findings during screening. Other exclusion criteria included known history of allergic reactions to MPH, and participation in another clinical trial within a period of 30 days prior to the first dose of study medication.

Eligible subjects were enrolled after signing informed consent documents. The clinical study protocol, informed consent documents and information forms were reviewed and approved by an Institutional Review Board (Institutional Review Board Services, Québec, Canada) and the Therapeutic Product Division (TPD) of Health Canada prior to beginning associated study procedures. The study was conducted in accordance with the Declaration of Helsinki and local regulatory requirements.

### Clinical procedures

For each study, 24 subjects were randomized and dosed with Sandoz MPH OCR (Sandoz Development Center Holzkirchen, Holzkirchen, Germany), and with Concerta® (Janssen-Cilag, Neuss, Germany), according to the randomisation schedule. Whole blood samples were collected prior to dosing, and over a period of 24 h following each dose. All blood samples were drawn into pre-chilled (ice-water bath) blood collection tubes (1 × 3 mL) containing ethylenediaminetetraacetic acid (EDTA) K_2_. After collection, blood samples were cooled in an ice bath, prior to being centrifuged at 3000 rpm (corresponding to approximately 1900 g) for at least 10 min at approximately 4°C. Two aliquots of at least 0.5 mL (when possible) of plasma were dispensed into polypropylene tubes (as soon as possible), containing a stabilizing solution (EDTA Na_2_ 10% w/v, citric acid 10% w/v in water), resulting in a buffer : plasma ratio of 5% v/v and were vortexed. The aliquots were subsequently transferred to a −80°C freezer, pending transfer to the bioanalytical facility.

### Bioanalysis

d-threo-methylphenidate and l-threo-methylphenidate plasma levels were both quantified using fully validated liquid chromatographic methods with tandem mass spectrometry (LC MS/MS). The method was developed by inVentiv Health Clinical, Montréal, Canada. The validation of the bioanalytical method followed international guidelines (FDA, 2001).

The analytes d-threo-methylphenidate and l-threo-methylphenidate and their internal standard methylphenidate-d9 were extracted from a 0.200 mL aliquot of human EDTA K_2_ plasma using an automated liquid–liquid extraction. The extracted samples were injected into a liquid chromatograph equipped with a Chirobiotic (V), 150 × 4.6 mm, 5 *μ*m column. The mobile phase was a mixture of methanol and acetic acid 0.15% (v/v) and ammonium hydroxide 0.05% (v/v). The isocratic chromatographic separation was performed at room temperature at a flow rate of 1.5 mL/min. The detection was made with a tandem mass spectrometry detector API 5000 (AB SCIEX, Concord, ON, Canada).

The lower limit of quantification of the analytical method for d-threo-methylphenidate was 0.15, 0.15, and 0.05 ng/mL for the 54, 36 and 18 mg doses, respectively. The lower limit of quantification of the analytical method for l-threo-methylphenidate was 6, 4, and 2 pg/mL for the 54, 36, and 18 mg doses, respectively. The upper limit of quantification of the analytical method for d-threo-methylphenidate was 30, 30, and 10 ng/mL for the 54, 36, and 18 mg doses, respectively. The upper limit of quantification of the analytical method for l-threo-methylphenidate was 600, 400, and 200 pg/mL for the 54, 36, and 18 mg doses, respectively. Plasma samples from subjects who did not complete the study were analysed and reported, but results were not used for statistical analysis. All bioanalytical work was conducted in compliance with Good Laboratory Practices.

### Pharmacokinetic evaluations

Pharmacokinetic assessment of bioequivalence was considered a suitable surrogate for efficacy evaluation. Evaluations were based on pharmacokinetic parameters (AUC_0–*t*_, AUC_0-inf_, *C*_max_, residual area: calculated as 100*(1 − AUC_0–*t*_/AUC_0–inf_), *T*_max_, *C*_max_, *T*_½el_, and *K*_el_) of d-threo-methylphenidate, which were calculated using Bioequiv (version 3.50, Anapharm, Montreal, QC, Canada) software, developed and tested for bioequivalence studies. The partial metrics AUC_0–2h_, AUC_2–24h_, *C*_max(0–2h),_ and *C*_max(2–24h)_ were also calculated for the fasting studies to evaluate the biphasic pharmacokinetic profiles of the two products.

### Safety and tolerability evaluations

All subjects who received a treatment dose were considered for the safety evaluation. Safety and tolerability evaluations were based on adverse event monitoring, physical examination, vital sign measurements (including oral temperature), and standard laboratory evaluation (haematology, biochemistry, endocrinology, and urinalysis).

### Statistical methods and determination of sample size

According to Hauschke et al. (1992), a sample size of 20 subjects was estimated to be sufficient to show bioequivalence (intra-subject coefficients of variation for AUC and *C*_max_ for d-methylphenidate: 21% or smaller; significance level: 5%; expected deviation from the reference: 5% (point-estimate); power: 80%) with an acceptance range for the 90% confidence interval of AUC_0-t_ and *C*_max_ within 0.80 and 1.25 for d-threo-methylphenidate. To account for possible dropouts, 24 subjects were enrolled in each study in order to complete with a minimum of 20.

For statistical analysis, only subjects completing both treatment periods were considered for pharmacokinetic evaluation. Administration of MPH extended-release tablets has been shown to result in plasma concentrations of the l-isomer of MPH of approximately 1/40th the plasma concentrations of the d-isomer (Janssen-Cilag 2013). Due to the low concentration and the resulting minor contribution of the l-isomer to the pharmacological activity, only the active d-isomer was evaluated for bioequivalence determination, in line with EMA guidelines on the investigation of bioequivalence (The European Medicines Agency 2010). All analyses of variance (ANOVAs) were performed with the SAS (release 8.2 for Windows) general linear models procedure (GLM). The ratio of means (Sandoz MPH OCR/Concerta®) and 90% geometric confidence interval for the ratio of means, based on least-squares means from the ANOVA of the ln-transformed data, were calculated for AUC_0–*t*_, AUC_0–inf_, and *C*_max_. *T*_max_ was reported descriptively. In accordance with the protocol, bioequivalence was based on the 90% geometric confidence interval of the ratio (Sandoz MPH OCR/Concerta®) of least-squares means from the ANOVA of the ln-transformed AUC_0–*t*_ and *C*_max_ falling within the range of 80% to 125% for d-threo-methylphenidate. In addition, ratio of means and 90% geometric confidence intervals were also calculated for the partial metrics AUC_0–2h_, AUC_2–24h_, *C*_max(0–2h),_ and *C*_max(2–24h)_ for the fasting studies, in order to also confirm bioequivalence for both the immediate- and extended-release phases.

## Results

### Subject disposition and baseline demographics

As per the protocol, in all four studies, 24 normal healthy volunteers were enrolled and randomized and were all eligible for the safety analysis. For the 36-mg dose fasting study, two subjects withdrew (one for personal reasons and one for failure to attend Period II) and were not eligible for pharmacokinetic analysis. In the 54-mg fed study, three subjects were withdrawn after Period I (one due to positive urine cotinine test at check-in for Period II, one was unable to attend Period II and one was withdrawn due to positive alcohol breath test at check-in for Period II); the remaining 21 subjects completed the study and were eligible for pharmacokinetic analysis. For the 54, 36, and 18 mg fasting and 54 mg fed studies, enrolled subjects had a mean age of 37, 33, 33, and 37 years and mean BMI of 24.2, 24.2, 24.0, and 23.8, respectively (Table1).

**Table 1:** Baseline demographics by study.

	54-mg fasting	36-mg fasting		18-mg fasting	54-mg fed	
Parameter (units) All mean ± SD	*n* = 24 (dosed subjects and study completers)	*n* = 24 (dosed subjects)	*n* = 22 (study completers)	*n* = 24 (dosed subjects and study completers)	*n* = 24 (dosed subjects)	*n* = 21 (study completers)
Age (years)	37 ± 11	33 ± 11	34 ± 11	33 ± 10	37 ± 11	36 ± 11
Weight (kg)	71.4 ± 6.7	75.3 ± 6.2	74.9 ± 5.8	74.2 ± 6.4	71.6 ± 6.9	71.5 ± 7.3
Height (cm)	171.8 ± 4.9	176.3 ± 6.0	175.8 ± 5.7	175.9 ± 7.0	173.4 ± 5.3	172.8 ± 5.0
BMI (kg/m^2^)	24.2 ± 1.9	24.2 ± 1.2	24.2 ± 1.3	24.0 ± 1.8	23.8 ± 1.6	23.9 ± 1.6

### Pharmacokinetics and statistical evaluation

Plasma samples from subjects who received any study medication were analysed and results reported. Samples from all subjects completing the study were included in the statistical analyses for d-threo-methylphenidate.

The pharmacokinetic parameters for Sandoz MPH OCR and Concerta® for each of the four studies are summarised in Tables2 and 3. The ratio of means (Sandoz MPH OCR/Concerta®) and 90% geometric confidence interval for the ratio of means, based on least-squares means from the ANOVA of the ln-transformed data are summarized in Tables4 and 5. Bioequivalence between the two formulations being compared was concluded if the 90% geometric confidence intervals of the ratio of least-squares means of Sandoz MPH OCR/Concerta® of ln-transformed AUC_0–*t*_ and *C*_max_ were within the acceptance range of 80% to 125%. As an example, the ratio for key pharmacokinetic parameters for the 18-mg fasting study were: 95.83% (90% CI: 92.99–98.76%) for AUC_0–*t*_, 96.35% (90% CI: 93.64–99.13%) for AUC_0–inf_, and 96.08 (90% CI: 89.24–103.44%) for *C*_max_. All other studies also met the bioequivalence criteria as 90% geometric confidence intervals for both AUC_0–*t*_ and *C*_max_ were within the predefined range.

**Table 2:** Descriptive statistics of pharmacokinetic parameters for Sandoz MPH OCR and Concerta® in the fasting studies.

Parameter (Units) All mean ± SD	54-mg fasting (*n* = 24)		36-mg fasting (*n* = 22)		18-mg fasting (*n* = 24)	
	Sandoz MPH OCR	Concerta®	Sandoz MPH OCR	Concerta®	Sandoz MPH OCR	Concerta®
AUC_0–*t*_ (ng.h/mL)	116.70 ± 26.52	121.43 ± 27.00	89.37 ± 34.31	94.92 ± 32.85	39.89 ± 10.23	41.54 ± 9.90
AUC_0–inf_ (ng.h/mL)	121.98 ± 28.07	125.36 ± 28.12	95.72 ± 38.98	99.91 ± 36.19	41.52 ± 10.64	43.07 ± 10.58
*C*_max_ (ng/mL)	10.89 ± 2.09	12.11 ± 2.95	7.94 ± 2.79	8.39 ± 2.61	3.62 ± 0.81	3.76 ± 0.77
*T*_max_ (h)	5.81 ± 1.01	6.96 ± 1.56	5.70 ± 1.04	7.32 ± 1.49	5.65 ± 0.76	6.42 ± 1.49
K_el_ (h^−1^)	0.1612 ± 0.0192	0.1811 ± 0.0199	0.1473 ± 0.0247	0.1634 ± 0.0211	0.1647 ± 0.0169	0.1781 ± 0.0200
*T*_½ el_ (h)	4.36 ± 0.55	3.88 ± 0.46	4.85 ± 0.93	4.32 ± 0.64	4.25 ± 0.43	3.94 ± 0.46
AUC_0–2 h_ (ng.h/mL)	6.59 ± 2.23	6.49 ± 2.18	4.93 ± 1.94	4.84 ± 1.51	2.26 ± 0.85	2.24 ± 0.83
AUC_2–24 h_ (ng.h/mL)	110.12 ± 24.85	114.93 ± 25.43	84.44 ± 32.96	90.08 ± 31.80	37.64 ± 9.64	39.29 ± 9.36
*C*_max (0–2h)_ (ng/mL)	5.43 ± 1.70	5.54 ± 1.86	4.00 ± 1.67	4.11 ± 1.24	1.88 ± 0.56	1.85 ± 0.57
*C*_max (2–24h)_ (ng/mL)	10.89 ± 2.09	12.11 ± 2.95	7.94 ± 2.79	8.39 ± 2.61	3.62 ± 0.81	3.76 ± 0.77

**Table 3:** Descriptive statistics of pharmacokinetic parameters for Sandoz MPH OCR and Concerta® in the fed study.

Parameter (units) All mean ± SD	54-mg fed (*n* = 21)	
	Sandoz MPH OCR	Concerta®
AUC_0–*t*_ (ng.h/mL)	141.02 ± 43.11	148.57 ± 47.73
AUC_0–inf_ (ng.h/mL)	148.92 ± 47.46	154.38 ± 51.61
*C*_max_ (ng/mL)	12.55 ± 3.36	13.35 ± 4.04
*T*_max_ (h)	5.17 ± 0.64	8.19 ± 2.64
K_el_ (h^−1^)	0.1599 ± 0.0341	0.1859 ± 0.0248
*T*_½ el_ (h)	4.52 ± 0.92	3.79 ± 0.52

**Table 4:** Ratio of means (Sandoz MPH OCR/Concerta®) and 90% confidence intervals based on least-squares means from the ANOVA of the ln-transformed data from the fasting studies.

Parameter (units) All mean ± SD	54-mg fasting (*n* = 24)		36-mg fasting (*n* = 22)		18-mg fasting (*n* = 24)	
	Ratio	90% geometric CI	Ratio	90% geometric CI	Ratio	90% geometric CI
AUC_0–*t*_	95.95%	92.68–99.33%	93.45%	90.27–96.73%	95.83%	92.99–98.76%
AUC_0–inf_	97.12%	94.12–100.21%	94.91%	91.51–98.44%	96.35%	93.64–99.13%
*C*_max_	90.96%	84.94–97.40%	93.69%	87.31–100.54%	96.08%	89.24–103.44%
AUC_0–2h_	100.29%	90.83–110.73%	99.29%	88.82–110.99%	100.30%	91.15–110.36%
AUC_2–24h_	95.67%	92.44–99.01%	93.03%	89.85–96.32%	95.58%	92.81–98.44%
*C*_max(0–2h)_	97.88%	90.95–105.33%	95.11%	86.36–104.74%	101.99%	94.28–110.33%
*C*_max(2–24h)_	90.96%	84.94–97.40%	93.69%	87.31–100.54%	96.08%	89.04–103.44%

Ratios calculated using least-squares means according to the formula: e^(treatment A (test) − treatment B (reference))^ × 100. 90% geometric confidence interval uses ln-transformed data.

**Table 5:** Ratio of means (Sandoz MPH OCR/Concerta®) and 90% confidence intervals based on least-squares means from the ANOVA of the ln-transformed data from the fed study.

Parameter (Units) All mean ± SD	54-mg fed (*n* = 21)	
	Ratio	90% geometric CI
AUC_0–*t*_	95.30%	90.26–100.62%
AUC_0–inf_	96.91%	92.12–101.95%
*C*_max_	94.88%	86.54–104.02%

Ratios calculated using least-squares means according to the formula: e^(treatment A (test) − treatment B (reference))^ × 100. 90% geometric confidence interval uses ln-transformed data.

**Figure 2:**
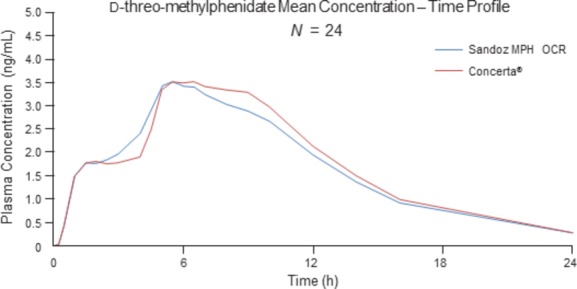
Mean plasma concentration of d-threo-methylphenidate over time, after administration of an 18-mg dose of Sandoz MPH OCR or Concerta® to healthy adult male subjects under fasting conditions.

Figure2 shows the mean plasma concentration of d-threo-methylphenidate over time, after administration of Sandoz MPH OCR and Concerta® for the 18-mg fasting study. The plasma concentration time curves under fasting conditions showed a biphasic profile, and were comparable with Concerta®. This was confirmed by bioequivalence of the partial metrics AUC_0-2 h_, AUC_2-24 h_, *C*_max(0-2h),_ and *C*_max(2–24h)_. The ratio for the partial pharmacokinetic parameters for the 18-mg fasting study were as follows: 100.30% (90% CI: 91.15–110.36%) for AUC_0–2h_, 95.58% (90% CI: 92.81–98.44%) for AUC_2–24h_, 101.99% (90% CI: 94.28–110.33%) for *C*_max(0-2h)_, and 96.08% (90% CI: 89.04–103.44%) for *C*_max(2-24h)_.

Under fed conditions, only approximately 50% of the profiles under both Concerta® and the Sandoz product were biphasic, whereas the other 50% of profiles showed a continuous increase. Therefore, separation of the immediate-release and extended-release phases was not possible.

### Safety and tolerability

In all studies both formulations were well tolerated, with no major side effects and no relevant differences in safety profiles with respect to the number and pattern of adverse events. A summary of the numbers of treatment-emergent adverse events (TEAEs) for each product is given in Table6. The most commonly reported TEAEs were “headache,” “palpitations” and “procedural site reaction.” Of the 74 TEAEs reported from the four studies, 47 were suspected to be related to the study medication. In the 54-mg fed study and the 36-mg fasting study, the number of subjects who experienced TEAEs following the administration of Concerta® was greater than the number experienced after administration of Sandoz MPH OCR, but this was not considered significant. The majority of TEAEs were mild; there were no serious adverse events or deaths in any of the studies.

**Table 6:** Summary of the number of common (≥2) treatment-emergent adverse events by product and study.

Treatment-emergent adverse event	54-mg fasting (*n* = 24)		36-mg fasting (*n* = 24)		18-mg fasting (*n* = 24)		54-mg fed (*n* = 24)	
	Sandoz MPH OCR	Concerta®	Sandoz MPH OCR	Concerta®	Sandoz MPH OCR	Concerta®	Sandoz MPH OCR	Concerta®
Cardiac disorders								
Palpitations			1	2			1	2
Gastrointestinal disorders								
Dry mouth								2
Injury, poisoning and procedural complications								
Procedural pain	1		2	1			1	1
Post-procedural swelling		1						2
Procedural site reaction		3		1				
Investigations								
Blood creatine phosphokinase increased					1			1
Heart rate increased				1	1			2
Metabolism and nutrition disorders								
Decreased appetite	1							1
Nervous system disorders								
Dizziness				1				1
Headache	2		2	3	1	1		1
Psychomotor hyperactivity							1	1
Somnolence				1		1		
Tremor			2	1				

## Discussion

Sandoz MPH OCR was found to be bioequivalent to Concerta® in terms of rate and extent of absorption when administered as a single dose of one extended-release tablet of 54, 36 or 18 mg under fasting conditions and at 54 mg under fed conditions. According to the relevant EMA Committee for Medicinal Products for Human Use (CHMP) guideline, bioequivalence can be established if the 90% confidence interval for the ratio Test/Reference of geometric least squares means for *C*_max_ and AUC_0–*t*_ falls within the acceptance limits of 80–125% (The European Medicines Agency 2010). Results from the studies confirmed that all pharmacokinetic parameters were clearly within the predefined acceptance limits. It is therefore possible to conclude that Sandoz MPH OCR is bioequivalent to Concerta® at doses of 54, 36, and 18 mg under fasting conditions and at a dose of 54 mg under fed conditions.

Concerta® exhibits a biphasic plasma concentration profile. This biphasic drug release is thought to prevent acute tolerance to MPH and to maintain efficacy throughout the day (Markowitz et al. 2003; Swanson et al. 2003). Sandoz MPH OCR was found to be equivalent to the OROS of Concerta®, as evidenced by the similar biphasic pharmacokinetic profile shown for the two products. The partial pharmacokinetic metrics for the fasting studies confirmed that Sandoz MPH OCR was bioequivalent to Concerta® in both the immediate- and extended-release phases. The cut-off of 2 h was chosen in line with EMA guidance (The European Medicines Agency 2013) according to the overall shape of the pharmacokinetic profile. Partial metrics using a cut-off of 3 h (AUC_0–3h_, AUC_3–24h_, *C*_max(0–3h)_ and *C*_max(3–24h)_) also demonstrated bioequivalence (data not shown).

Under fed conditions, only approximately 50% of profiles are biphasic for both Sandoz MPH OCR and Concerta®. The remaining profiles show a continuous increase, which means that drug release from the immediate-release and extended-release phase overlap and cannot be separated by a cut-off; hence the immediate-release and the extended-release phase cannot be adequately characterized using partial pharmacokinetic parameters under fed conditions (Modi et al. 2000). The two different types of profiles (biphasic or continuous) under fed conditions are most likely caused by differences in the first site of deposition of the dosage form within the stomach directly after ingestion. If the tablet is deposited in the antrum of the stomach, the dissolved drug from the immediate-release phase can bypass the stomach contents and is quickly transported to the intestine where is can be absorbed. In this case, a biphasic profile can be observed. On the other hand, if the tablet is deposited in the fundus of the stomach, the dissolved drug from the immediate-release phase is trapped by the high-fat meal in the stomach and only reaches the intestine with delay. Absorption only takes place at a later time when drug is already released from the extended-release phase of the dosage form. Thus, absorption from the two phases overlaps, resulting in a continuous profile. Such behaviour has been demonstrated for a biphasic amoxicillin/clavanulate fixed-dose combination. (Weitschies et al. 2008) It should be noted that, despite the differences in the shape of the pharmacokinetic profiles, the products can be taken under both fasting and fed conditions. (Janssen-Cilag 2013; Sandoz 2011).

Both products were well tolerated and no relevant differences in the safety profiles were observed between Sandoz MPH OCR and Concerta®. There were no serious adverse events or deaths in any of the studies.

## Conclusion

Sandoz MPH OCR was shown to be bioequivalent to Concerta® in terms of rate and extent of absorption when administered as a single dose of one extended-release tablet of 54, 36, or 18 mg under fasting conditions and at a dose of 54 mg under fed conditions.

## Abbreviations

ADHD: attention deficit/hyperactivity disorder
ANOVA: analyses of variance
BMI: Body Mass Index
EMA: European Medicines Agency
GLM: general linear models procedure
LC MS/MS: liquid chromatographic methods with tandem mass spectrometry
MPH: methylphenidate
OCR: osmotic-controlled release
OROS: osmotic-controlled-release oral delivery system
TEAEs: treatment-emergent adverse events
TPD: Therapeutic Product Division
